# Reelin gene therapy promotes cognitive resilience in a mouse model of amyloid induced tauopathy

**DOI:** 10.1002/alz.092691

**Published:** 2025-01-03

**Authors:** Dylan Finneran, Qingyou Li, Aurélie Joly‐Amado, Taylor Desjarlais, Brianna Jackman, Marcia N. Gordon, Kevin Nash, Dave Morgan

**Affiliations:** ^1^ Michigan State University, Grand Rapids, MI USA; ^2^ University of South Florida, Tampa, FL USA; ^3^ Michigan Alzheimer’s Disease Research Center, Ann Arbor, MI USA

## Abstract

**Background:**

A recent study of familial Alzheimer’s disease identified a mutation in the *RELN* gene that appeared to delay the onset of dementia. It was hypothesized that this *RELN‐COLBOS* variant protected against dementia by enhanced signaling at reelin receptors. We previously developed a secreted, bio‐active reelin fragment (R36) and packaged it into AAV. Recently, we found that intravenous AAV‐tau delivered in capsids (AAV.B10) that cross the blood brain barrier led to increased tauopathy in APP+PS1 (A+P) transgenic mice compared to nontransgenic (NonTg) littermates.

**Method:**

Nine‐month old mice were intravenously injected with AAV.B10 expressing P301L 2N4R human tau to produce A+P^TAU^ and NonTg^TAU^. Concurrently, these mice were injected intracerebroventricularly (ICV) with AAV9 expressing either R36 or green fluorescent protein (GFP). Learning and memory was assessed by radial arm water maze (RAWM) four months after surgery. Untreated A+P and NonTg mice were included as controls. RIPA soluble and insoluble hippocampal fractions were assayed for total tau, phospho‐tau, Aβ40, Aβ42, and oligomeric Aβ by ELISA.

**Result:**

All NonTg mice, regardless of tau treatment, learned and correctly recalled the RAWM platform location. Recall in untreated A+P mice was slightly impaired. A+P^TAU^ mice given ICV‐GFP performed at chance levels on all trials. In contrast, A+P^TAU^ mice given ICV‐R36 performed significantly better than ICV‐GFP A+P^TAU^ mice, but not as well as untreated A+P mice, indicating a partial rescue of the tau‐associated memory deficit by ICV‐R36. Tauopathy, as measured by ELISA, was unchanged by R36 over‐expression, but insoluble Aβ42 was reduced in ICV‐R36 A+P^TAU^ mice compared to ICV‐GFP A+P^TAU^ mice.

**Conclusion:**

We conclude that ICV injections of AAV expressing a bio‐active fragment of reelin can partially protect against cognitive impairment in a mouse model of amyloid induced tauopathy. This is consistent with a potential protective role of reelin signaling in Alzheimer’s disease. While we did not observe an effect of R36 over‐expression on tau pathology, we do demonstrate a significant reduction of insoluble Aβ42. We continue to investigate additional chemical and histological markers in these mice to better understand the protective influence of reelin.

